# Focused ultrasound mitigates pathology and improves spatial memory in Alzheimer's mice and patients

**DOI:** 10.7150/thno.79898

**Published:** 2023-07-14

**Authors:** Maria Eleni Karakatsani, Robin Ji, Maria F. Murillo, Tara Kugelman, Nancy Kwon, Yeh-Hsing Lao, Keyu Liu, Antonios N. Pouliopoulos, Lawrence S. Honig, Karen E. Duff, Elisa E. Konofagou

**Affiliations:** 1 Department of Biomedical Engineering, Columbia University, New York, USA.; 2 Taub Institute for Research on Alzheimer's Disease and the Aging Brain, Columbia University Irving Medical Center, New York, USA.; 3 Department of Neurology, Columbia University Irving Medical Center, New York, USA.; 4 UK Dementia Research Institute, University College London, London, UK.; 5 Department of Radiology, Columbia University Irving Medical Center, New York, USA.

**Keywords:** Alzheimer's Disease, Focused Ultrasound, Blood-Brain Barrier Opening, Amyloid beta and Tau, Drug-free therapy

## Abstract

**Rationale:** Bilateral sonication with focused ultrasound (FUS) in conjunction with microbubbles has been shown to separately reduce amyloid plaques and hyperphosphorylated tau protein in the hippocampal formation and the entorhinal cortex in different mouse models of Alzheimer's disease (AD) without any therapeutic agents. However, the two pathologies are expressed concurrently in human disease. Therefore, the objective of this study is to investigate the effects of repeated bilateral sonications in the presence of both pathologies.

**Methods:** Herein, we investigate its functional and morphological outcomes on brains bearing both pathologies simultaneously. Eleven transgenic mice of the 3xTg-AD line (14 months old) expressing human amyloid beta and human tau and eleven age-matched wild-type littermates received four weekly bilateral sonications covering the hippocampus followed by working memory testing. Afterwards, immunohistochemistry and immunoassays (western blot and ELISA) were employed to assess any changes in amyloid beta and human tau. Furthermore, we present preliminary data from our clinical trial using a neuronavigation-guided FUS system for sonications in AD patients (NCT04118764).

**Results:** Interestingly, both wild-type and transgenic animals that received FUS experienced improved working memory and spent significantly more time in the escape platform-quadrant, with wild-type animals spending 43.2% (sham: 37.7%) and transgenic animals spending 35.3% (sham: 31.0%) of the trial in the target quadrant. Furthermore, this behavioral amelioration in the transgenic animals correlated with a 58.3% decrease in the neuronal length affected by tau and a 27.2% reduction in total tau levels. Amyloid plaque population, volume and overall load were also reduced overall. Consistently, preliminary data from a clinical trial involving AD patients showed a 1.8% decrease of amyloid PET signal 3-weeks after treatment in the treated hemisphere compared to baseline.

**Conclusion:** For the first time, it is shown that bilateral FUS-induced BBB opening significantly and simultaneously ameliorates both coexistent pathologies, which translated to improvements in spatial memory of transgenic animals with complex AD, the human mimicking phenotype. The level of cognitive improvement was significantly correlated with the volume of BBB opening. Non-transgenic animals were also shown to exhibit similar memory amelioration for the first time, indicating that BBB opening results into benefits in the neuronal function regardless of the existence of AD pathology. A potential mechanism of action for the reduction of the both pathologies investigated was the cholesterol metabolism, specifically the LRP1b receptor, which exhibited increased expression levels in transgenic mice following FUS-induced BBB opening. Initial clinical evidence supported that the beta amyloid reduction shown in rodents could be translatable to humans with significant amyloid reduction shown in the treated hemisphere.

## Introduction

Alzheimer's disease is the most common age-related and chronic neurodegenerative disorder characterized histopathologically by the accumulation of insoluble forms of amyloid-β (Aβ) in plaques and the aggregation of hyperphosphorylated forms of the microtubule-associated protein-tau into neurofibrillary tangles (NFT) in neurons 1,2. Extensive research reports that these two hallmark lesions, and the accumulation of toxic intermediates from which they are formed, are implicated in the initiation and progression of dementia. Aβ is cleaved from amyloid precursor protein (APP) into a group of peptides of varying length (between 38 and 43 amino acids) and slightly different characteristics 3,4. Proteolytic studies have demonstrated that Aβ is a normal product of APP metabolism and is generated at high levels in neurons, but also by other cell types 1. The neuronal function of APP remains unknown, but it might be involved in synaptic plasticity. Aβ accumulation and conformational changes that lead to a high βsheet structure is central in AD pathogenesis 5. The pathological cascade of events that leads to tauopathy and neurodegeneration includes tau hyperphosphorylation, misfolding and aggregation, destabilization of the microtubule network and the cytoskeleton, deficits in axonal transport, synaptic loss, neurodegeneration and cell death. Tauopathy has been shown to propagate between brain regions, and it can undergo trans-cellular transfer, either from the somatodendritic compartment or at the axon terminals across the synapses 6. The mechanism of transfer is not known, but it is likely vesicle-mediated or via the release and uptake of conformers that act as prion-like seeds 6,7. Multipronged approaches have been developed to understand the underlying causes of AD, including proteomics and transcriptomics, to identify key biomarkers for not only diagnostic purposes, but also for therapeutic development 8-10.

One such biomarker of interest is Apolipoprotein E (ApoE). Cholesterol homeostasis in the brain is inevitably bound to the production, transport and function of ApoE, the strongest genetic risk factor for AD, especially since its polymorphisms in the transcriptional regulatory region have been associated with the disease 11. Cholesterol is the principal element for the nervous system, since the brain contains 20% of the entire body's cholesterol 12. It is a major structural component of the cell membranes as well as myelin sheaths and is tightly regulated between neurons and glia cells 12-14. The BBB prevents lipoprotein permeation from the circulation, thus mandating the de novo synthesis of cholesterol in the brain primarily by oligodendrocytes followed by astrocytes and then neuronal somas 12-14. Astrocytic cholesterol suffices to cover functional needs of the neurons that are involved in cholesterol turnover 13,14, while about 1% of excess cholesterol can be stored in an esterified form, known as lipid droplets 13. Cholesterol homeostasis is essential for brain functioning given that cholesterol is involved in hormonal synthesis, synaptic and dendritic formation, axonal guidance and cell function both during development and adult stage 13. ApoE is highly expressed in the brain primarily by astrocytes and microglia and is involved in the normal catabolism of lipoproteins 11. As a result, Aβ, tau and ApoE are three elements that have substantial evidence as contributors of AD 11.

Ultrasound-facilitated BBB opening has been evaluated in a battery of clinical trials recruiting initially Glioblastoma multiforme (GBM) patients. The completion of the trial revealed an increase in the median progression-free survival in the 19 enrolled patients, who received repeated treatments prior to systemic administration of carboplatin 15. These beneficial outcomes paved the way for the expansion of clinical research utilizing ultrasound leading, eventually, to the first clinical trial revealing a safe and reversible BBB opening in 5 AD patients using MR-guided ExAblate system (Insightec Inc., Tirat Carmel, Israel) 16. Although a secondary outcome measure, researchers evaluated the amyloid load in the patients' brains prior to and following the sonication without detectable changes. Providing the initial feasibility of the study, a phase IIa clinical trial was designed and is currently enrolling AD patients who will be repeatedly sonicated for three times in total (NCT03739905). Another clinical trial (NCT03671889) employing a similar MR-guided system is actively selecting AD patients to be embedded in a study with safety and efficacy as the primary outcome measures. Currently, 28 out of 132 clinical trials administering pharmacological agents to AD patients reached the phase III stage, 11 investigating symptomatic agents - three cognitive enhancers and eight alleviating behavioral symptoms- and 17 disease-modifying drugs -six biological and eleven small-molecule therapies. In the disease-modifying therapeutic strategies, 10 are primarily, if not exclusively, targeting amyloid-β, one tau pathology and the rest are neuroprotective, anti-inflammatory or metabolic approaches 17. However, the ultimate objective of the clinical research in sonicating AD patients without delivering a pharmacological agent lies in the replication of the promising pre-clinical data that indicate pathological amelioration accompanied by functional improvement 18,19. Recently, our in-house developed clinical system for neuronavigation-guided focused ultrasound (FUS) induced BBB opening outside the magnetic bore was approved to treat AD patients including assessment of pathological alteration following the procedure (NCT04118764) 20 and some initial findings are presented herein.

The primary advantage of FUS-mediated BBB opening alone is the ability to non-invasively modulate the innate immune response in a targeted region 21,22, which has been implicated in the pathological amelioration and functional improvements in AD models. Briefly, over the past decade it was proven that amyloid plaques can be reduced following repeated ultrasound targeted in one region or swept across the brain 18,19,23. One prominent mechanism for the amyloid clearance from the brain is that FUS can directly trigger the innate immune response and activate microglia, since microglia have been found to engulf the amyloid plaques following sonication 19,24. Moreover, a time course study investigating the effect and persistence of a single sonication in amyloid plaques, concluded that the plaque volume reduced to more than half their initial one, the days following sonication and the effect persisted for two weeks 25. Therefore, repeated, biweekly sonications were evaluated and indicated a plaque population reduction on the order of 27% accompanied by a 40% decrease in surface area 25. Preclinical findings targeting amelioration of tau pathology by FUS-induced BBB opening have also recently been reported. The studies conducted so far, though employing different transgenic animal models overexpressing mutant human tau, converge on their primary findings that phosphorylated tau can be significantly reduced following repeated sonications 26-30. The activation of the immune response to the sonication and autophagy have been proposed as potential mechanisms of tau clearance 26-28. Despite the promising preclinical evidence, a common limitation in these studies lies in the expression of a single pathology in the animal models employed. Animals overexpress proteins linked to familial AD (FAD), mutant amyloid precursor protein (APP), or APP and presenilin (PS), resulting in the formation of amyloid plaques 31. Alternatively, rodents would overexpress a mutation (P301L or K369I) in the microtubule-associated protein tau (MAPT), resulting in destabilization of the microtubule network and tauopathies 26-29. However, these models fall short in mimicking human AD that is defined by the presence and interplay of both amyloid plaques and neurofibrillary tangle pathology. Therefore, the beneficial outcomes and the proposed mechanisms of these studies need to be evaluated in animal models with concurrent pathology similar to human disease.

We examine herein whether Alzheimer's pathology can be mitigated by ultrasound to (i) investigate the efficacy of repeated bilateral FUS for the reduction of human total tau and amyloid plaques from the 3xTg-AD mice that display both plaque and tangle pathology in the hippocampus, (ii) establish the functional imprint of the pathological amelioration attributed to FUS module by Morris Water Maze (MWM) assessment and (iii) determine the interplay of pathological changes with cholesterol trafficking 32-35. Finally, initial findings from an AD human subject that underwent FUS in the amyloid-affected brain region are also reported.

## Results

### Experimental study

In this study, forty-four (N = 44) animals (transgenic and non-transgenic) were included in total to assess pathological differences and functional outcomes attributed to the effect of pathological alteration and ultrasound application. Additionally, one mild AD subject was treated with FUS as part of our phase I clinical trial (NCT04118764) and the amyloid PET signal was measured over time. The patient satisfied the trials' inclusion criteria, which includes a diagnosis of early/moderate Alzheimer's disease or mild cognitive impairment, a mini mental state examination (MMSE) score between 12 and 26, and a positron emission tomography (PET) scan confirming amyloid plaque load.

### FUS-induced BBB opening did not differ in volume between groups and across weeks

**Figure 1:**
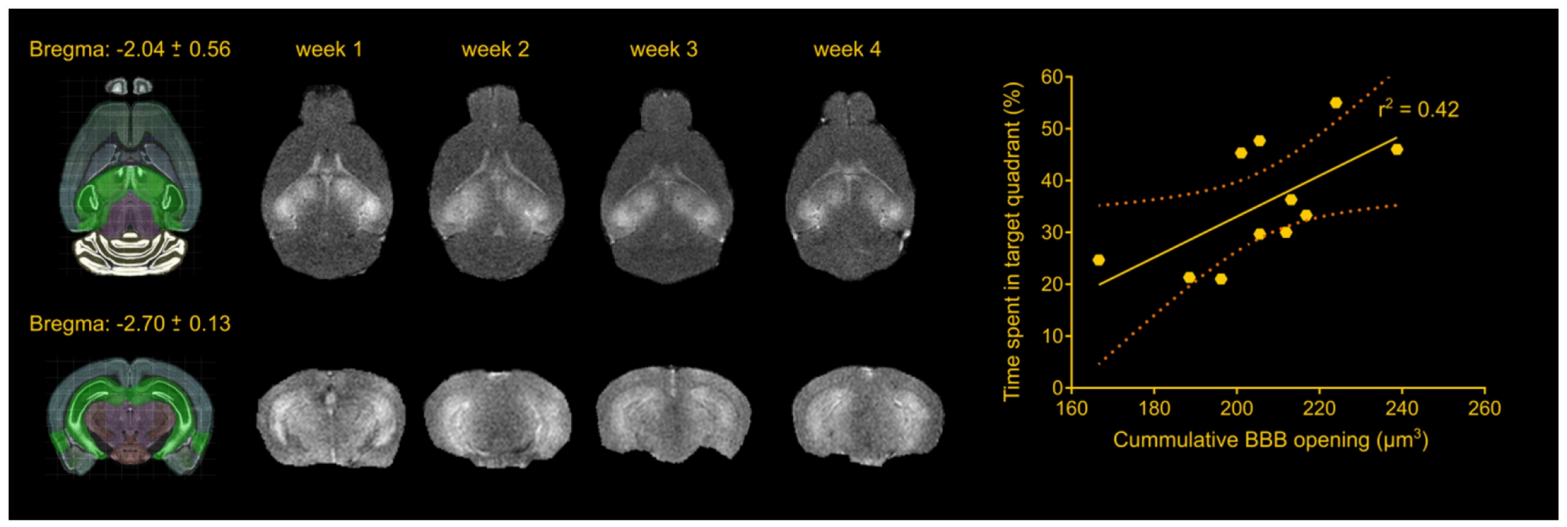
Repeatability of FUS-induced BBB opening and association with behavioral outcomes in the transgenic animals. The BBB opening volume was measured on the order of 57.5 ± 8.3 mm^3^, 51.4 ± 12.5 mm^3^, 51.00 ± 9.3 mm^3^ and 46.3 ± 14.2 mm^3^ for the four weeks respectively. Statistical analysis revealed comparable opening sizes across weeks. Regression analysis between the cumulative BBB opening volume and the time spent in the target quadrant showed a linear relationship (r^2^ = 0.42) with a significant deviation of the slope from the zero value (slope = 0.3941; F[1.9] = 6.43; P = 0.0319). Brain schematics were constructed using Brain Explorer 2 provided by Allen Brain Atlas.

The foremost important aspect of the study was the repeatability of the BBB opening using a cavitation controller system and the bilateral targeting accuracy in this transgenic mouse model. Magnetic resonance imaging revealed consistency in the BBB opening among samples as well as longitudinally across weeks (Figure 1), similar to our previous studies 22,30. Quantification of the BBB opening volume in the transgenic animals was measured on the order of 57.5 ± 8.3 mm^3^, 51.4 ± 12.5 mm^3^, 51.00 ± 9.3 mm^3^ and 46.3 ± 14.2 mm^3^ for the four weeks respectively. Statistical analysis confirmed the lack of significant differences in the opening size across weeks, consistent with our previously reported results 22,30, deeming repeated ultrasound suitable for this transgenic mouse model at this age given the technique did not compromise the barrier or initiated any edematous incidences as indicated by the T2-weighted imaging.

### Spatial memory improvement in the transgenic cohort following sonication

**Figure 2:**
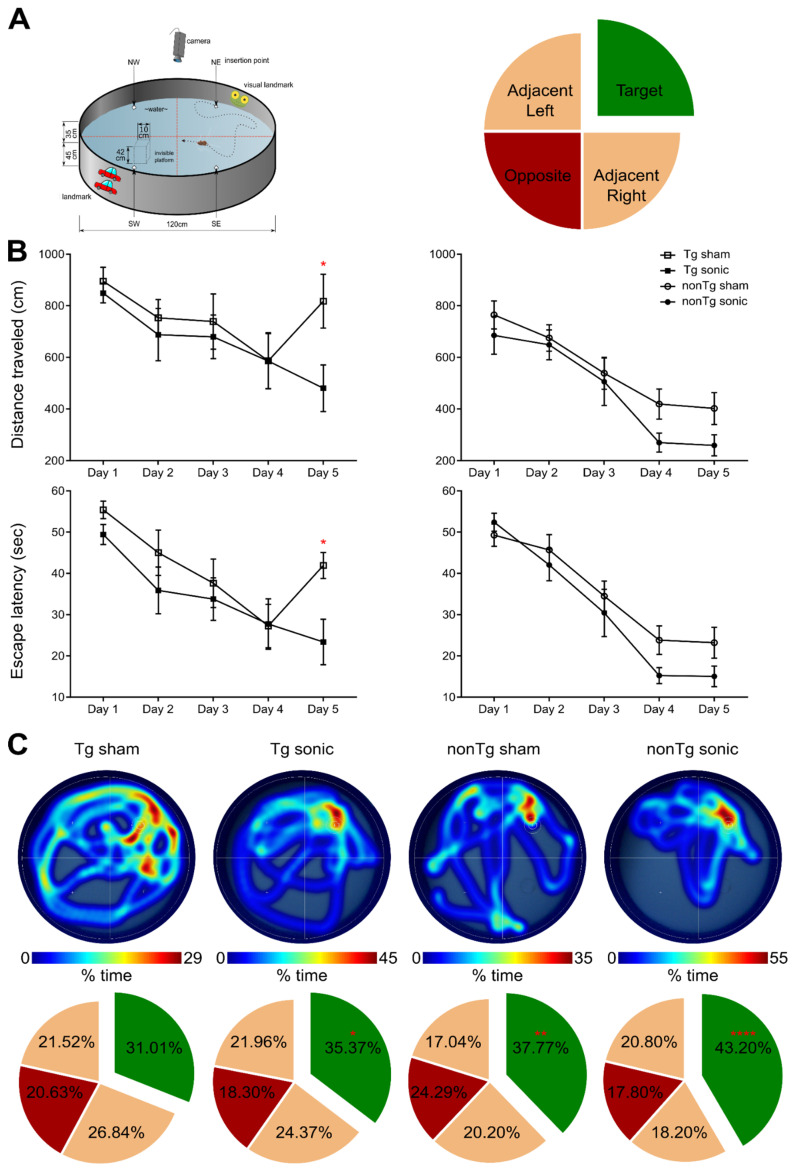
Repeated sonications improve spatial memory in transgenic and non-transgenic mice: (A) The schematic of the Morris Water Maze setup that was used to behaviorally examine the animals in respect to their spatial memory functionality. The circular maze is considered to consist of four quadrants, the target, the opposite and the adjacent. (B) Learning curves of the escape latency and the distance traveled to find the hidden platform are presented herein and plotted against the days of training. Both the distance traveled (F[4,144] = 15.57; P < 0.0001) and the escape latency (F^3^,^36^ = 7.542; P = 0.0005) significantly deteriorated for the sham transgenic animals the last day of the training while all other groups continued to improve. (C) Representative individual heatmaps of the time spent in every quadrant followed by the cumulative results on the probe trial. The sham transgenic animals did not show preference over any quadrant while the sonicated transgenic animals spent significantly more time in the target quadrant (F[1.637, 16.37] = 4.051; P = 0.044). Respectively the non-transgenic mice spent more time in the target quadrant with the sonicated group at a much higher percentage (sham non-transgenic: F[1.965, 17.68] = 6.375; P = 0.0085, sonicated non-transgenic F[1.776, 15.99] = 16.48; P = 0.0002).

Consistency in the BBB opening location and magnitude was of primordial importance to accurately assess behavioral changes associated with the learning rate and short-term spatial memory with contained variance. MWM has been established to detect cognitive changes related to learning and memory given the confinement of the amyloid aggregation and tangle accumulation in the hippocampus, cortex and amygdala, in this specific strain 33. MWM examines the functionality of the long- and short-term memory that should be significantly impaired at this age (15-16 month-old) since the impairment is evident starting at 6 months of age 32,34. To investigate the effect of ultrasound in the cognitive changes detected, transgenic and non-transgenic mice underwent the same task and three metrics were employed: the escape latency from the maze, the distance traveled from the dropping point to the escape platform and the time spent in each quadrant following the platform removal (probe trial). Mice received four training trials per day for five consecutive days in the spatial version of the MWM shown in the schematic of Figure 2A. In regards to the distance traveled, an effect was observed on both days (F[4,144] = 15.57; p < 0.0001) and groups (F^3^,^36^ = 7.542; p = 0.0005) indicating a differentiation in the learning pace (Figure 2B). The sonicated transgenic group reached the escape platform traveling similar distance as the non-transgenic groups (sham and sonicated), while the sham transgenic group traveled significantly longer distances until reaching the platform. Equivalent results were obtained for the escape latency with a significant effect in days (F[4,144] = 35.71; p < 0.0001) but not for groups, indicative of the resulting learning improvement for all groups over the training days with similar learning rates (Figure 2B). Despite the comparable learning pace, the sham transgenic group significantly deteriorated on the fifth day, needing more time to escape the maze, while the sonicated transgenic group remained consistently fast at escaping, similar to the sham non-transgenic and sonicated non-transgenic groups. Two hours after the training trial, a 60 s probe trial was conducted during which the animals were allowed to spend time in the maze without a hidden platform and the time spent in the target, opposite and adjacent quadrants was measured. Representative heatmaps from each group show the percent of time spent in the maze followed by the cumulative results (Figure 2C). The sonicated transgenic mice spent significantly longer time in the target quadrant compared to the opposite (target:35.37%, opposite:18.30%, adjacent left:21.96%, adjacent right:24.37%; F[1.637, 16.37] = 4.051; p = 0.044), in contrast to the sham transgenic animals that spent comparable amount of time in every quadrant (target:31.01%, opposite:20.63%, adjacent left:21.52%, adjacent right:26.84%). These results indicate that FUS-mediated BBB opening resulted in partially rescuing spatial memory deficits in the transgenic animals.

### Spatial memory improvement in the non-transgenic mice following sonication

The non-transgenic cohort was included in the behavioral study to serve as the control group which, according to their performance, drove the number of training days. The last day of the training was thus determined as the day the non-transgenic animals achieved equivalent escape latency to the previous day (fifth day).

Apart from regulating the experimental conditions, the non-transgenic animals would also provide essential information as to whether any observed cognitive changes could be attributed to FUS alone and not to changes in the Alzheimer's-related protein load in the brain. During the training sessions, non-transgenic groups (sham and FUS groups) traveled comparable distance at similar latencies across all days. However, the percent of time the sham non-transgenic animals spent in the target quadrant was found significantly higher than the opposite (target: 37.77%, opposite: 24.29%, adjacent left: 17.04%, adjacent right:20.20%; F[1.965, 17.68] = 6.375; p = 0.0085), while the sonicated non-transgenic group spent significantly more time in the target quadrant compared to all other quadrants (target: 43.20%, opposite: 17.80%, adjacent left: 20.80%, adjacent right: 18.20%; F[1.776, 15.99] = 16.48; p = 0.0002). This finding is of potential formidable importance since it indicates that the treatment improved the short-term memory performance of non-transgenic mice. Taken together and given the beneficial effect of ultrasound on spatial memory established by the improved performance of the sonicated non-transgenic group, it remains unclear whether the improved functionality observed in the sonicated transgenic mice could be attributed to the ultrasound effect alone or combined with reduction in pathology. Interestingly, regression analysis between the cumulative BBB opening volume integrated over the four sonications and the time spent in the target quadrant indicated a linear relationship between variables (r^2^ = 0.42) with a significant deviation of the slope from the zero value (slope = 0.3941; F[1.9] = 6.43; p = 0.0319) as shown in Figure 1. Thus, cumulative BBB opening, as a proxy of repeated sonications and the resulting downstream effects, could potentially predict the behavioral outcomes in terms of short-term spatial memory, a finding that needs to be further explored.

### Tau reduction follows FUS-induced BBB opening

**Figure 3:**
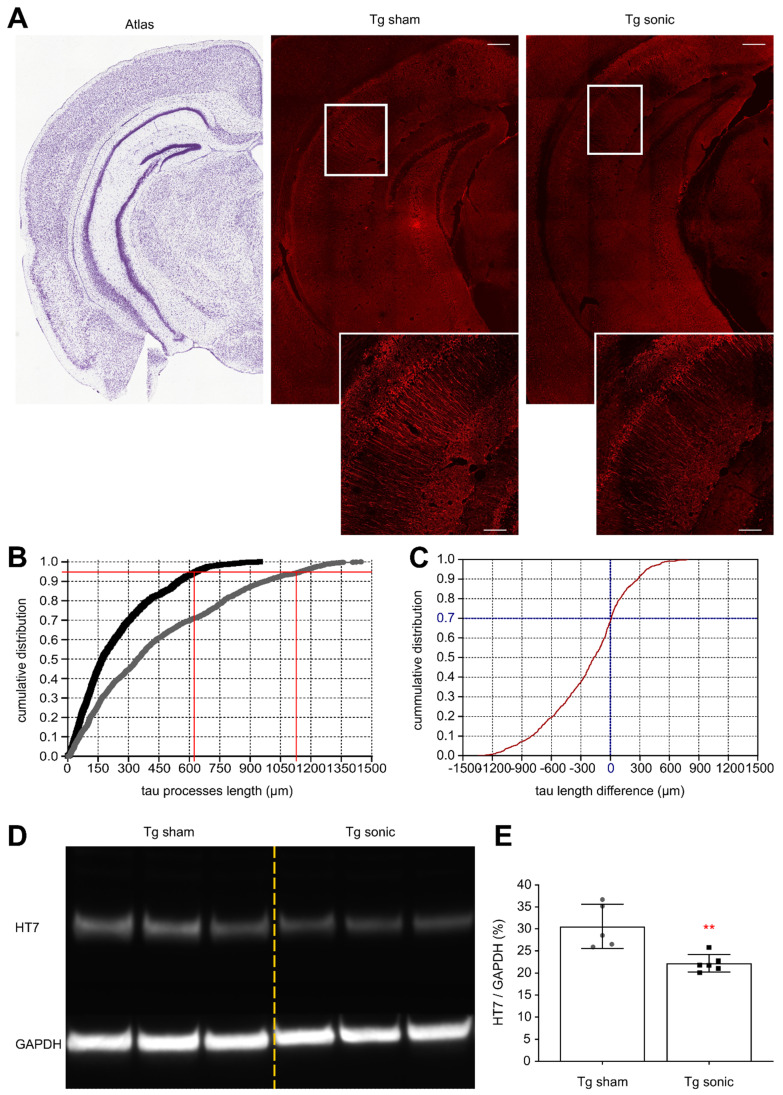
Human total tau quantification. (A) Representative examples of the tau signal emitted from the affected CA1 pyramidal neurons of the sham and sonicated brains (atlas, sham hemisphere, sonicated hemisphere). Scale bar, 100 μm. Immunohistochemical analysis of the hHT7 antibody detecting human total tau protein revealed a decrease of the neuronal processes affected by tau on the order of 49.6% (t^7^ = 5.013; P = 0.0015). (B) Comparison of the cumulative density function (CDF) of the tau processes length as obtained from each group. The graph describes the probability (y-axis) of finding tau processes of a certain or smaller length (x-axis). The 95th percentile (horizontal red line) crosses the CDF of the sonicated brain at 620 μm (left vertical red line), while at 1150μm (right vertical red line) for the sham brain. Thus, there is a 95% probability to find a tau neuronal process equal or smaller than 620 μm in the sonicated brain and 1150 μm in the sham brain. (C) The cumulative density function of the difference in tau processes length between the sonicated and sham hemispheres (sonicated-sham). From this CDF it can be observed that 70% of the neurons sonicated tau-affected neurons are shorter than those on the sham brain. (D) - (E) Tau removal from the hippocampus shown by immunoblot analysis. Taking the ratio of the HT7 over the GAPDH average intensity-band showed a significant reduction in the brains treated with ultrasound compared to the sham group on the order of 27.33% (t^9^ = 3.771; P = 0.004).

To determine whether the ameliorative effects of FUS in the brain with isolated tau pathology observed in previous studies 28,30 could be observed when amyloid concurrently develops in the hippocampus, immunohistochemistry (IHC) analysis and immunoblotting were performed. Evident from the confocal images (Figure 3A) is the reduction of human total tau (HT7) protein from the neuronal processes similar to our previous findings 30. Quantification of the processes' length using the structural algorithm developed herein revealed a significant reduction of the neuronal length affected by tau on the order of 49.6% (t^7^ = 5.013; p = 0.0015). In Figure 3B, the cumulative density function (CDF) demonstrates the tau processes length across the two groups. The 95^th^ percentile, denoted by the horizontal red line, crosses the CDF of the sonicated brains at 620 μm (left vertical red line), while at 1150 μm (right vertical red line) for the sham brain. This finding indicates that there is a 95% probability to find a tau neuronal process equal or smaller than 620 μm in the sonicated brain and 1150 μm in the sham brain. Similarly, the CDF of the length difference in tau processes between the sonicated and sham hemispheres is shown in Figure 3C. The probability of the sonicated tau processes being smaller than the sham tau process is almost 70% (zero crossing of the CDF). Tau reduction from the hippocampus was confirmed by immunoblot analysis as shown in Figure 3D-E. Taking the ratio of the HT7 over the GAPDH average intensity-band showed a significant reduction in the brains treated with FUS compared to the sham group on the order of 27.33% (t^9^ = 3.771; p = 0.004).

### Aβ-42 and Aβ-40 reduction follows FUS-induced BBB opening

**Figure 4:**
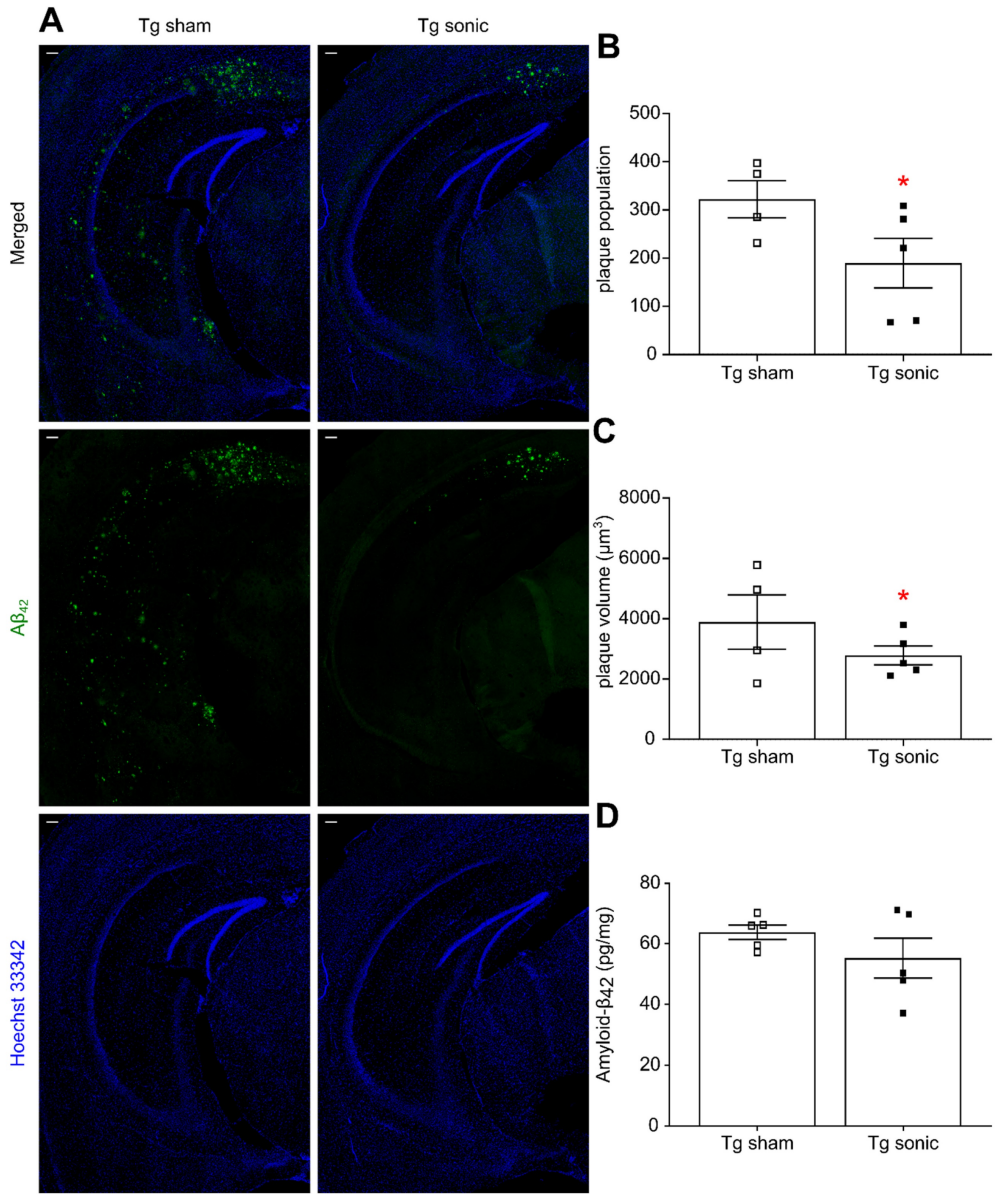
Amyloid plaque quantification. (A) Aβ immunoreactivity was examined using the Αβ42 specific antibody used in both immunohistochemistry and ELISA while Hoechst 33342 was employed for anatomical navigation. Plaque quantification revealed a decrease in the (B) population and (C) volume on the order of 51.39% (t^7^ = 2.707; P = 0.0303) and 42.23% (t^7^ = 2.835; P = 0.0252), respectively, in the sonicated brains. (D) Along the same lines, Αβ42 quantification with sandwich ELISA confirmed the decreasing trend following sonications 9.33%, yet lacking significance (t^9^ = 0.851; P = 0.41). Scale bar: 100μm.

Following behavioral testing, animals were sacrificed, and their brains either processed for immunohistochemistry or homogenized for quantification. Aβ immunoreactivity was qualitatively found less pronounced in the transgenic mice that received sonications (Figure 4A) as evidenced by the presence of the Aβ-42 specific antibody. Plaque quantification revealed a significant decrease in the population (Figure 4B) on the order of 51.39% (t^7^ = 2.707; p = 0.0303) and the volume (Figure 4C) on the order of 42.23% (t^7^ = 2.835; p = 0.0252) in the brains that repeatedly received ultrasound. Similarly, Aβ immunoreactivity evidenced by the presence of the Aβ-40 specific antibody (Figure S1A) revealed a 24.79% (t^7^ = 2.871; p = 0.024) plaque volume decrease and a 47.41% (t^7^ = 2.584; p = 0.0363) population reduction in the sonicated brains (Figure S1B-C). The plaque reduction observed in the IHC quantification readouts was confirmed by an overall Αβ-42 and Αβ-40 load reduction on the order of by 9.33% (t^9^ = 0.851; P = 0.41, Figure 4D) and 12.42% (t^9^ = 1.289; p = 0.2335, Figure S1D), respectively, yet lacking significance, as quantified by ELISA.

### Cholesterol metabolism-associated genes are upregulated in sonicated transgenic brains

**Figure 5:**
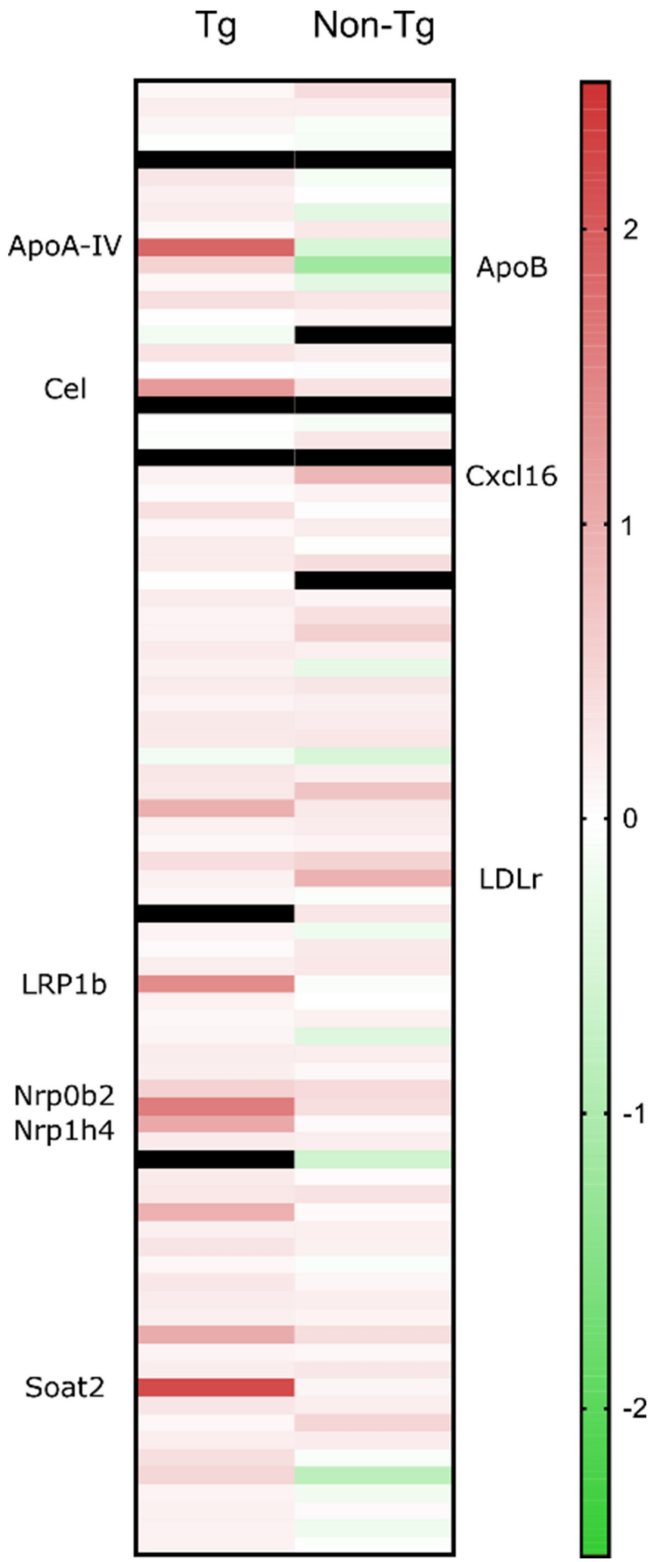
Quantitative real time PCR results are presented as the fold change of the sonicated brain normalized to the sham brain. From the cholesterol-related genes, the significant elevations in the transgenic animals' mRNA involved those encoding LRP1b and cholesterol homeostatic regulators including lipoprotein ApoA-IV, nuclear receptor subfamilies, Nr0b4 and Nr1b4, cholesterol esterification and cholesteryl hydrolysis enzymes, Soat1 and Cel and endothelial scavenger receptor Scarf1. For the non-transgenic animals, the significant gene upregulations involved, LDLr and the scavenger receptor for oxidized low density lipoproteins, chemokine (C-X-C motif) ligand 16 (CXCL16) while ApoB, the main LDLr ligand, was the only downregulated gene.

Isolated hippocampi from the left hemisphere were stored in RNA preservative upon extraction prior to transcriptomic analysis. Real-time quantitative PCR (RT-qPCR) showed that 24 hours following sonication, several cholesterol-related genes were significantly upregulated (> 2-fold) compared to the sham group. Increased cholesterol synthesis in the brain has been previously reported in mice with disrupted BBB 36. The elevations in mRNA involved genes (Figure 5) encoding LRP1b and cholesterol homeostatic regulators including lipoprotein ApoA-IV, nuclear receptor subfamilies, Nr0b4 and Nr1b4, cholesterol esterification and cholesteryl hydrolysis enzymes, Soat1 and Cel and endothelial scavenger receptor Scarf1 37. Of the upregulated genes, LRP1b is of immense importance as it shares 50% of its amino acid sequence with LRP1 and was initially described as a putative tumor suppressor 38. According to the literature, protein domain structure comparison of the two genes proved an almost identical structural organization of the two proteins strongly suggesting similar ligand interactions 39 However, two major structural differences translated to functional variations with the LRP1b exhibiting a much slower rate of endocytosis influencing the cellular distribution and catabolism of ligands 40. Increased levels of APP have been detected at the surface of cells overexpressing LRP1b suggesting that the receptor can alter susceptibility to AD by modulating APP processing and reducing Aβ peptide production 40. Additionally, LRP1 is the main receptor for Aβ transport across the BBB in the direction of brain to blood and peptides with a great β-sheet content, such as Aβ42, bind with great affinity to the receptor 41. Moreover, genetic silencing of LRP1 almost completely blocked the uptake of tau, characterizing LRP1 as the master regulator in tau uptake and spreading 42. In contrast, enhanced tau ligation and neuronal distribution was not observed for the LRP1b or the other low-density lipoprotein receptor (LDLr) family members 42. Collectively, upregulation of LRP1b receptor following sonication could be implicated in the reduction of Aβ42 production by prolonged binding to APP as well as transport of Aβ42 to the circulation. In regards to tau pathology, it is currently unknown how LRP1 could be advantageous in reducing tau from the brain but it has been proven that the receptor does not increase spreading by neuronal intake 42. Considered together with the current literature, we propose the implication of the LRP1b receptor in the clearance of tau concurrent with the reduction in Aβ_42_.

### Cholesterol metabolism-associated genes are upregulated in sonicated non-transgenic brains

To further investigate the implication of ultrasound in cholesterol trafficking, we sonicated the left hippocampus of non-transgenic animals, transcriptomically analyzed with the same cholesterol profiler kit. RT-qPCR showed that 24 hours following sonication, the cholesterol-related genes that significantly increased (> 2-fold) compared to the sham group include LDLr and the scavenger receptor for oxidized low density lipoproteins, chemokine (C-X-C motif) ligand 16 (Figure 5). The mechanisms underlying the neuroprotective activity of CXCL16 require the interaction of microglia, astrocyte and neurons and the activity of the Adenosine receptor type 3 (A3R), with consequent release of CCL2 by glial cells. Such an interaction has been reported to occur in mice and rats following single or repeated sonications 43,44. The only significantly downregulated gene was ApoB, the main LDLr ligand, other than ApoE. The downregulation of ApoB could be attributed to the intake of lipoprotein from the upregulated receptor. Moreover, it has been previously shown that in the absence of LDLr, hippocampal impairment and cognitive decline were experienced in mice that underperformed in MWM probe trial where they spent significantly less time in the target quadrant 45. Therefore, LDLr upregulation is proposed to trigger the performance of the FUS group spatial memory, since spatial memory retention has been achieved in an APOE-LDLr dependent pattern spatially localized in the hippocampus 46. Further investigation of this interplay is warranted to robustly conclude on the role of the LDL family in memory retention and pathology ameliorations previously described.

### Amyloid reduction following FUS is translatable to an AD patient

**Figure 6:**
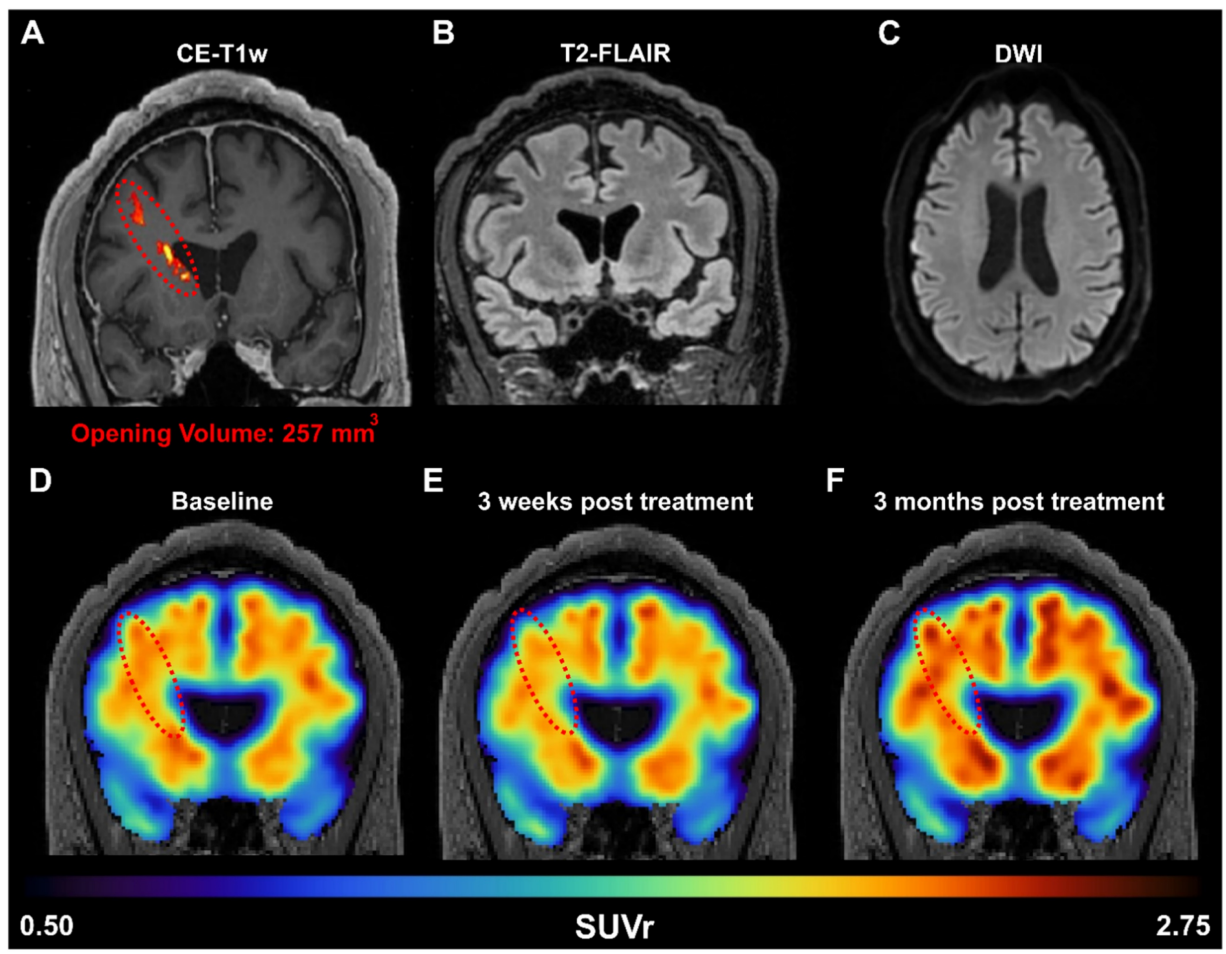
(A) Amyloid PET signal reduction following FUS treatment in an early AD patient. BBB opening in the right prefrontal cortex was confirmed immediately after FUS exposure through contrast enhanced T1-weighted (CE-T1w) MRI, revealing a contrast enhancement volume of 257 mm^3^ (red dotted oval). MRI safety scans, acquired on the day of treatment, including (B) T2-FLAIR and (C) DWI, indicate a safe opening without edema or hemorrhaging. The amyloid PET signal was measured at the (D) baseline pre-FUS, (E) 3 weeks, and (F) 3 months post-FUS. Within the treated frontal lobe, we detected a reduction of 1.8% in the SUVr 3 weeks after treatment, followed by a 5.9% increase in SUVr 3 months after treatment, all relative to the baseline scan.

To confirm whether the FUS-induced amyloid reduction detected in rodents could be translated to humans, we performed a targeted unilateral BBB opening in the prefrontal cortex of AD patients in a phase I clinical trial (NCT04118764). An example of one patient (Sex: Male, Age: 60 years old, mini mental state examination (MMSE) score: 18/30) is shown in Figure 6A. A 257 mm^3^ BBB opening was observed in a contrast-enhanced T1-weighted MRI scan 2 hours after FUS treatment. Additionally, T2-FLAIR (Figure 6B) and DWI (Figure 6C) sequences were acquired on the same day and did not present any hypo- or hyper- intensities, indicating the absence of acute edema or hemorrhaging and demonstrating that FUS induced BBB opening was well tolerated. Quantification of the standardized uptake value ratio (SUVr) within the treated frontal lobe showed a 1.8% reduction in SUVr 3 weeks after treatment (Figure 6E) but a 5.9% increase in SUVr 3 months after treatment (Figure 6F), when compared to the baseline SUVr acquired during screening. For reference, the contralateral left frontal lobe had a SUVr reduction of 0.4% at 3 weeks and a 6.7% increase at 3 months. The observed reduction in SUVr (i.e., amyloid-beta load) in the frontal lobe 3 weeks after FUS-induced BBB opening suggests that the observed preclinical findings in transgenic mice may be translatable to humans as well. However, the reduction in SUVr was not persistent at 3 months post-treatment, which may indicate that FUS-induced reduction of amyloid-beta load is a transient response, and treatment programs may require multiple sessions in human patients. Furthermore, unilateral sonications were only investigated in patients, while the preclinical data presented herein investigated bilateral sonications in transgenic mice. Future work in patients investigating the efficacy of bilateral sonications is warranted, however initial proof-of-concept and potential translatability is demonstrated in a patient with AD.

## Discussion

Overall, we showed herein that both hallmarks of AD pathology, human Aβ42 and human tau protein, could be mitigated by through weekly application of FUS-mediated BBB opening. In addition to the significant reduction in tau from the hippocampal formation, the spatial distribution of the pathological marker altered following BBB opening. Neuronal processes were less affected by tau that was significantly reduced from the brain, evidenced by immunoblotting. Concurrent with the beneficial outcomes in tau distribution, the amyloid plaque population, volume, and overall load were also significantly decreased, yet the effect was less pronounced, and not statistically significant, when an immunological assay (ELISA) was employed to assess amyloid load. Ultimately, ultrasound may have mitigated the pathology progression and resulted in functional improvement and especially in the enhancement of short-term spatial memory. However, it is still unclear whether the pathological amelioration or its synergy to the downstream effects following sonication are responsible for the behavioral outcomes. Significant improvement in the memory of non-transgenic animals after repeated sonications opens avenues for further investigation of the effects FUS has on memory cells. Behavioral measures following FUS have been previously shown in alert and healthy non-human primates, whose reaction time improved by 23 ms and touch error reduced by 0.76 mm while being sonicated 47. Shen *et al*. previously demonstrated similar findings in reduction of both amyloid-beta and tau tangles in 3xTg mice that translated to behavioral changes (e.g. learning and memory) following repeated unilateral openings 48. Despite the promising findings shown herein, additional experiments may be necessary to disentangle the effect of repeated FUS from the pathology mitigation in the memory improvement. Behavioral measures including open field to establish the effect of FUS on reduction of anxiety and novel object identification (NOI) to study the effect of amelioration of memory impairment caused in AD mice models as a FUS protective mechanism, should be evaluated. Moreover, the duration of the behavioral response to the number and the time course of related FUS treatment should be studied to enable recommendations for future treatments.

Proteomic analysis was also performed, highlighting 20 different proteins that were expressed only in mice receiving FUS-induced BBB opening. Our results presented here complement prior findings and provide quantitative (Western Blot and ELISA) evidence in the reduction of both hallmarks of AD using a cavitation controller system for multiple bilateral sonications. It is the first-time tau reduction has been reported and confirmed by both immunoblotting and immunohistochemical image analyses in the brains that suffer from concurrent amyloid and tau pathology. Furthermore, transcriptomic analysis of the sonicated hippocampi revealed upregulation of the LDLr family (Figure 5), closely associated with cholesterol trafficking in the brain and memory retention, highlighting a potential mechanism of action for the observed reduction the AD pathology in mice receiving FUS-induced BBB opening. The LDLr proteins are structurally homologous and exhibit various important endocytic and signaling functions 49 whereas among the seven members of the family, the LDLr and the LDLr-related protein 1 (LRP1 and LRP1b) are the main receptors for the uptake of ApoE proteins in the brain 14. In brief, LDLr is mainly expressed in glia cells and binds extracellularly to Apolipoprotein B (ApoB) and ApoE 50. LDLr deficiency leads to learning difficulties, impaired spatial cognition, ApoE increase 51 and amyloid deposits while overexpression of LDLr has been associated with decreased ApoE levels and amyloid inhibition 52. Genomic research suggests that LDLr is one of a few cholesterol genes associated with the risk of developing AD 53 while LDLr deficient patients showed a high incidence of mild cognitive impairment 54. LRP1 is involved in signaling pathways, vascular protection, regulation of cell migration and BBB integrity modulation 55. In particular, LRP1 mediates Aβ uptake in astrocytes and neurons followed by subsequent lysosomal degradation while mice with LRP1 deficient neurons developed exacerbated amyloid pathology) 56,57. It has also been proposed that LRP1 regulates Aβ uptake in glial cells by controlling inflammatory responses and phagocytic machinery 58. Finally, it has been shown that endothelial cell lines can internalize and degrade Aβ through LRP1 59,60. Thus, LRP1 appears positioned to modulate the levels of amyloid and, in so doing, possibly to regulate the progression of AD 61.

Preliminary evidence provided herein supports that the amyloid reduction as a result of FUS is translatable to humans. After a single sonication in the right frontal lobe, there was decreased amyloid PET signal in the patient's right frontal lobe 3 weeks after FUS-induced BBB opening, demonstrating beneficial short-term changes. However, this reduction was only temporary, as on the long-term, the amyloid PET signal increased again, indicating that the reduction is transient and that repeated treatments may be necessary for significant therapeutic outcomes. Furthermore, it is important to note that the percentage of BBB opening in the mouse was orders of magnitude greater than that in human subjects (approximately 10% in mice vs. < 0.1% in human) and in different regions of the brain targeted (bilateral hippocampus targets in mice vs. unilateral prefrontal cortex target in human). Additionally, unilateral sonications were only investigated in patients, while the preclinical data presented herein investigated bilateral sonications in transgenic mice. Future work in patients investigating the efficacy of bilateral sonications is warranted, however initial proof-of-concept and potential translatability is demonstrated in a patient with AD. These differences in treatment regime, opening volume, and BBB opening targets could explain the differences in the degree of therapeutic benefits observed between mice and humans.

The initial findings from our study add to the growing literature that FUS-induced BBB opening in patients with AD is not only safe and effective, but can also induce reduction in amyloid-beta load in patients 16,62-64. Epelbaum *et al.* employed an ultrasound device implanted within the skull (SonoCloud-1) to repeatability open the BBB in the supra-marginal gyrus in patients with mild AD over a 3.5 month period and observed an average SUVr reduction of 6.6% and 5.7% at 4 month and 8 month after the start of the trial 62. Other studies utilize the magnetic resonance-guided FUS (MRgFUS) system (InSightec ExAblate) for BBB opening, which are performed within the MRI bore but does not require a skull implant. D'Haese *et al.* opened the BBB in the hippocampus and entorhinal cortex (EC) in AD patients over three sessions (once every two weeks), and reported an average decrease in SUVr of 6.46% in the treated hippocampus/EC, relative to baseline, 7 days after the last sonication (approximately 5 weeks after first sonication) 63. Interestingly, 2 out of their 6 patients also observed reduction in SUVr in the contralateral hippocampus/EC. Furthermore, Park *et al.* performed bilateral sonications in the frontal lobe of AD patients over two sessions (once every three months) and found a significant reduction in SUVr (-1.6%, relative to baseline) 3 months after the last sonication 64. In contrast, Lipsman et al. opened the BBB in the frontal lobe as well but did not observe significant changes in amyloid-beta load in patients 1 week following BBB opening 16. The clinical FUS system used herein was an in-house developed system where we employed a neuronavigation system in conjunction with a low-frequency single-element transducer to be able to perform non-invasive BBB opening in patients outside of the MRI bore 20,65,66. At this point, it is clear that FUS-induced BBB opening can be achieved safely and reversibly in patients with AD. Our and others' findings on Aβ load reduction following BBB opening are consistent with the preclinical findings we presented herein. However, the long-term effects on amyloid-load, and potentially tau tangles, in patients need to be better elucidated. The treatment regimen (i.e., brain target, opening frequency, opening volume) in the AD patients has yet to be optimized and may explain the variability in findings across groups. Future studies using our neuronavigation-guided system covering a larger region of the brain across multiple sessions may yield a larger and more sustainable reduction in amyloid-beta load. Furthermore, therapeutics, ranging from small molecules 67-70 to neurotrophic factors and antibodies (e.g., Aducanumab) 71, which have proven to exert therapeutic effects in the context of AD, may work synergistically to ameliorate the pathological hallmark of AD.

This study demonstrated that repeated FUS-mediated BBB opening in the hippocampus was capable of reducing the two hallmarks of AD, amyloid beta and tau tangles, in the 3xTg-AD mouse model. The observed reduction in Aβ and tau translated to functional improvements in spatial memory, suggesting repeated FUS-induced BBB opening could rescue cognitive deficits. A potential mechanism of action for the reduction of the both pathologies could constitute cholesterol metabolism, specifically the LRP1b receptor, which exhibited increased expression levels in transgenic mice following FUS-induced BBB opening. Furthermore, initial clinical results with a neuronavigation-guided FUS system to open the BBB in a mild AD patient demonstrated apparent short-term reduction amyloid-beta load, which did not persist three months after opening, thus warranting repeated sonications for sustained reduction in pathology as confirmed by the animal findings. Future studies are thus required to better interpret this reduction in AD patients, as well as investigate whether tau reduction equally translates to patients. In conclusion, the findings we presented herein reinforce the efficacy of FUS-induced BBB opening as an alternative, non-invasive, drug-free approach in simultaneous reduction of the hallmark proteins of amyloid beta and tau tangles in Alzheimer's Disease.

## Materials and Methods

### Ethics Committee Approval and Patient Consent

All animal procedures presented herein were approved by the Columbia University Institutional Animal Care and Use Committee. All procedures involving human subjects were reviewed and approved by the Columbia University Irving Medical Center Institutional Review Board. Each participant enrolled in the study provided written consent to participate in this study. This study was registered to ClinicalTrials.gov under the number identifier NCT04118764.

### *In vivo* experiments

For this study, 22 female mice of the 3xTg-AD line at the age of 14 months were employed. This widely used strain contains three mutations associated with familial Alzheimer's disease (APP Swedish, MAPT P301L, and PSEN1 M146V) and expresses a progressive development of both plaques and tangles 33. Age- and gender-matched non-transgenic animals were included in the study as well, while all animals were randomly assigned to the sham and sonicated groups (eleven mice per group). For transcriptomic analysis a second cohort of 10 female mice of the 3xTg-AD line (14 months old) along with age- and gender-matched non-transgenic animals was incorporated. Animals were group-housed under standard conditions (12 h light/dark cycles, 22^o^C), were provided with a standard rodent chow (3 kcal/g; Harlan Laboratories, Indianapolis, IN, USA) and water ad libitum.

The animals involved herein (sham and sonicated) were anesthetized with a mixture of oxygen and 1-2% isoflurane (SurgiVet, Smiths Medical PM, Inc.,WI), placed prone with the head immobilized by a stereotaxic apparatus (David Kopf Instruments, Tujunga, CA) and depilated to expose the suture anatomy and minimize acoustic impedance mismatch. Identification of the parietal and interparietal bone intersection enabled proper positioning of the transducer following a grid-guided targeting procedure 72. Targeting the hippocampal formation involved positioning of the transducer 3 mm anteriorly to the lambdoid suture, and 1.5 mm laterally to cover the dorsal part, while 2 mm anteriorly and 3.2 mm laterally for the ventral part. Both hemispheres were targeted in this study in alternating order in consecutive sessions.

The mice included in the treatment groups (transgenic and non-transgenic) received FUS in a cavitation-controlled regime 22 with a pulse length of 10 ms and a repetition frequency of 5 Hz at PNP of 0.40 MPa after accounting for 18% murine skull attenuation. Acoustic emissions were monitored in real time using a cavitation controller system to assess the degree of cavitation and animals were injected with microbubbles intravenously until the cumulative cavitation dose reached a pre-set level associated with a single BBB opening on the order of 31.3 ± 16 mm 22. Thus, to accommodate for a constant cavitation level, the microbubble administration during the sonication ranged between 4-6 intravenous injections of polydisperse in-house manufactured 73,74 microbubbles diluted in saline (0.1 μl/g, mean diameter: 1.4 μm), while the duration of a single sonication varied from 8 to 360 sec. Reinjections were administered when the cavitation levels dropped below 1 dB, relative to the baseline cavitation levels when no microbubbles were present. The sham groups were subjected to the same procedure without triggering the transducer, received the average microbubble injections, as established by the sonicated groups and remained under anesthesia the average sonication duration. All animals underwent magnetic resonance imaging for BBB opening confirmation and safety assessment. This procedure was repeated once per week for four consecutive weeks for the first cohort. Following the last sonication, all animals were transferred to the behavioral facility to acclimate for five days prior to behavioral testing. Behavioral testing using the MWM followed and lasted for five consecutive days. After the last day of behavioral testing, which was approximately three weeks after the last sonication, the mice were sacrificed at the age of 15-16 months. The second cohort underwent the same ultrasound treatment only once and were sacrificed 24h following the sonication.

### Focused ultrasound

The sonications were carried out by a single-element, spherical-segment FUS transducer operating at 1.5 MHz, driven by a function generator (Agilent, Palo Alto, CA, USA) through a 50-dB power amplifier (E&I, Rochester, NY, USA). The central void of the therapeutic transducer held a pulse-echo ultrasound transducer (center frequency: 10 MHz, focal depth: 60mm, radius 11.2 mm; Olympus NDT, Waltham, MA) used for alignment, with their two foci coaxially aligned. The imaging transducer was driven by a pulser-receiver (Olympus, Waltham, MA, USA) connected to a digitizer (Gage Applied Technologies, Inc., Lachine, QC, Canada). A cone filled with degassed, distilled water was mounted onto the transducer assembly. The transducers were attached to a computer-controlled 3D positioning system (Velmex Inc., Lachine, QC, Canada).

### Magnetic resonance imaging

All animals underwent magnetic resonance scanning with the 9.4T MRI system (Bruker Medical, Boston, MA) following the sonication procedure. The mice were placed in a birdcage coil (diameter 35 mm), while being anesthetized with 1 - 2% isoflurane and respiration rate was monitored throughout the imaging sessions. MR images were acquired using a contrast-enhanced T1-weighted 2D FLASH sequence, 30 minutes following the intraperitoneal bolus injection of 0.2 ml gadodiamide (GD-DTPA) (Omniscan, GE Healthcare, Princeton, NJ) while T2-weighted MRI was performed one day after the sonication to detect any potential damage using a 2D RARE sequence 75.

### Morris Water Maze (MWM)

In order to test spatial learning and short-term memory, MWM was conducted, following the procedure previously described 35. The test was carried out during the light cycle and in a circular pool of 120 cm in diameter, filled 45 cm with tap water. The water was opaque by the addition of nontoxic white paint (Crayola) and kept at room temperature throughout. The pool contained spatial cues placed along the wall, which were kept constant through all testing days (Figure 2A). The trials were recorded and analyzed using WaterMaze video tracking software (Actimetrics).

The training was a five consecutive day acquisition period, made up of four trials per day. Each animal was assigned a specific target quadrant, consistent through all trials and days, which contained an escape platform. The platform was consistently kept 3 cm below the surface of the water, hidden from the animals during the test. The dropping-quadrant for each animal was alternated in a semi-random order and it would be either opposite or adjacent to the target quadrants; the assigned target quadrant was never selected as the dropping-quadrant (Figure 2A).

Each trial began when the animal was gently lowered into the water, facing the pool's wall. Each animal was allowed to find the platform within 60 sec. If the animal did not reach the platform within the given timeframe, it was then guided and gently placed on the platform. The animal was allowed to remain on the platform for 15 sec before being removed. After each trial the animal was placed in a cage lined with absorbent material and allowed to rest for 60 sec under a heat lamp. On the fifth day a probe trial was carried out, which occurred 3 hours after the last training trial. The hidden platform was removed from the target quadrant during the probe trial and the animal was lowered into the quadrant opposite to the target quadrant. It was given 60 sec of exploration time before being removed. This probe trial tests the acquisition of spatial learning and short-term memory 32,34,35,76. The behavioral measures that were recorded and analyzed were the latency to reach the platform, the distance traveled during the training trials and the time spent in every quadrant during the probe trial (Figure 1).

### Immunofluorescence

Five animals per group (5/11) were transcardially perfused (30 mL PBS followed by 60 mL 4% paraformaldehyde) following the completion of behavioral testing. The brains were extracted, preserved in PFA for 24 h and then in 30% Sucrose for at least 2 days prior to freezing. After brains were frozen on dry ice, cryostat-cut coronal hippocampal sections (35 μm) were collected in anti-freezing solution. Then, at least 3 free-floating sections of the intermediate and caudal hippocampus with a 6-sections gap were processed for immunohistochemistry. Prior to staining brain sections were pre-treated with 70% Formic Acid for 3 minutes to expose the Aβ42 epitope. The sections were sequentially washed in 0.1M PBS, treated with 5% donkey serum in 0.3% PBST for thirty minutes with the addition of mouse seroblock (BUF041, Bio-Rad Laboratories) for the last ten minutes followed by overnight incubation at 4°C with the primary antibodies: i. mouse anti-human-tau (HT7, 1:500, (HT7, 1:500, MN1000, Invitrogen) and rabbit anti-human-Aβ42 (1:500, AB5078P, EMD Millipore) antibodies. Then, sections were washed in PBS and incubated for 60 min in 0.3% PBST with 5% donkey serum containing the respective donkey-raised secondary antibodies, Texas Red- (1:1000, SAB3701155, Sigma-Aldrich) and Alexa488-conjugated (1:1000, SAB4600036, Sigma-Aldrich). After the incubation, the sections were washed again in PBS and mounted on slides. The slides were washed three times with 0.02% Tween20 in PBS and treated with Hoechst 33342 (5 μm/ml, H21492, Thermo Fisher) for ten minutes. Finally, the slides were washed with PBS and cover slipped with Fluoromount-G (00-4958-02, Thermo Fisher) solution.

A similar IHC procedure was followed for the identification of the Aβ40 epitope with using only the rabbit anti-human-Aβ40 (1:500, AB5078P, EMD Millipore) primary antibody in the overnight incubation and the Alexa488-conjugated (1:1000, SAB4600036, Sigma-Aldrich) secondary antibody for fluorescent labelling.

The large field confocal images of the hippocampus were captured on a 20X objective of a Nikon confocal microscope (Nikon Instruments Inc., Melville, NY, USA) with the same exposure parameters for the lasers. Tile (mosaic) and a Z-stack (2 μm step, 7 series) were necessary were acquired and processed to produce the resulting maximum intensity image.

### Immunoassays

Six animals per group (6/11) were sacrificed by cervical dislocation with their brains extracted and the hippocampus dissected on ice within a minute prior to storage at -80°C. Frozen hippocampi were weighed and homogenized without thawing in T-PER buffer (78510, Thermo Fisher) containing a protease and phosphatase inhibitor cocktail (78442, Thermo Fisher). The homogenate was then split into two parts and stored at -80°C until used for immunoblots and enzyme-linked immunosorbent assays (ELISA).

The first part of the homogenates was centrifuged at 3,000 g for 10 min at 4°C and the supernatant was recovered. Proteins were run on 4-12% Bolt Bis-Tris precast gels (NW04120BOX, Invitrogen) using 1 × MES SDS running buffer (B000202, Invitrogen) and under reducing conditions (B0009, Invitrogen). Proteins were then transferred to nitrocellulose membranes (iBlot2, IB23001, Invitrogen) using iBlot™ 2 Gel transfer system (IB21001, Invitrogen). Blots were blocked for 60 min using 5% non-fat dry milk (Omniblok, Americanbio Inc) in Tris-buffered saline with Tween-20 (TBST, 0.1M Tris, 0.15M NaCL and 0.1% Tween-20). Blots were then incubated overnight at 4°C in 5% milk TBS-T with HT7 primary antibody (1:2000, MN1000, Invitrogen) and loading control GAPDH (1:20000, G8795, Sigma-Aldrich). The next day blots were washed with TBST and then incubated for 1 h at room temperature in 5% milk TBST with HRP conjugated secondary antibody (1:100000, 115-035-003, Jackson ImmunoResearch Laboratories Inc.). Membranes were developed with enhanced chemiluminescent solution (SuperSignal™ West Pico Plus ECL, 34580, Thermo Scientific) and imaged using FluorChem imaging system (Alpha Innotech Corp). Protein band intensities were then quantified using ImageJ software (NIH, Bethesda, United States). The average intensity of the human total tau (HT7) band over the average intensity of the control band (GAPDH) of the immunoblot were quantified and the results are presented as the HT7/GAPDH ratio.

The second part of the homogenates was ultracentrifuged at 100,000 g for 1 h at 4°C. Supernatant was then recovered and used to measure soluble Aβ-42 and Aβ-40 levels using ELISA. The measurements were carried out using the specific Human Aβ-42 and Aβ-40 kits (KHB3441, Invitrogen) following the manufacturer's included protocol.

### Quantitative Real-time PCR

Real-time PCR was used to quantify the level of cholesterol related mRNA in the brain. One day after FUS-induced BBB opening, mice were sacrificed by cervical dislocation the left hippocampus extracted and stored in RNA preservative. The analysis was performed with a RNeasy Plus Mini Kit (Qiagen, Hilden, Germany). The mRNA levels were measured by qPCR using the SYBR Green assay (Applied Biosystems, Carlsbad, CA, USA). Signals from the hippocampi, sonicated and sham were compared, and fold changes were obtained.

### Quantification Algorithms

#### MR Volumetry

The enhancement in the horizontal plane of the MR images was quantified by volumetric analysis encompassing the hippocampal formations in both hemispheres. 3D images were normalized with the average reference-intensity obtained from the volume of an out-of-focus structure enhancing the contrast of the Gadolinium-diffuse area. Active contours were employed 77 to isolate the hyperintense area corresponding to the BBB openings on every slice throughout the entire brain by matching the number of slices to the size of the geometric beam according to the acquired resolution. The sum of the pixels enclosed within the contours yielded the area of the BBB opening while performing the analysis over 17 consecutive slices, resulted in the BBB opening volume reported herein 78.

### Confocal image analysis

The hippocampal formation was isolated from the surrounding brain tissue utilizing the merged-channel images (composite), thus constructing a hippocampal mask. For individual biomarker analysis, the masked composite maximum intensity images were split into the constituent channels. Throughout the rest of this study, the red and green channels represent signal emitted from human total tau and Aβ42, respectively.

The structural algorithm previously developed by our group was employed herein to quantify the number of cells and the length of the processes affected by tau 30. In brief, the hippocampal mask was constructed and applied onto the red channel followed by k-means segmentation to isolate the highest intensities. The Hough transform enabled the detection of circular objects and the identification of the pathological cell centers. Morphological operators connected the neighboring pixels and skeletonization, revealed the backbone of the processes, the branches and the endpoints. Utilizing the cell center coordinates as the initial point, the algorithm followed the skeleton within a defined neighborhood until reaching all endpoints. The distance of every endpoint to the cell center was measured and the longest 5% of the paths leading to the same cell were measured and averaged. The Monte Carlo simulation constructs the probability density function (PDF) for the process length of each group (sonicated and sham) followed by random sampling from each PDF and pairwise subtraction of the random samples 30. The subtraction is demonstrated as the cumulative density distribution revealing the percentage of length difference.

For the estimation of the amyloid plaque volume and population resulting from image quantification was implemented by utilizing the green channel. The entire image was normalized by the average signal emitted from an out-of-focus region, thus enhancing the contrast of the plaques with the surrounding tissue, followed by binarization with a fixed threshold throughout the analysis. The boundaries of the plaques were automatically detected 79, the area enclosed within each boundary measured and the volume inferred by the resolution and the in-plane radius of each plaque in MATLAB. The algorithm aside from the plaque volume yielded the location and the population per image.

### Clinical study

An AD subject (Sex: Male, Age: 60 years old, mini mental state examination (MMSE) score: 18/30) was treated in the right prefrontal cortex with neuronavigation-guided FUS 20 as part of our phase I clinical trial (NCT04118764). The study inclusion criteria included a diagnosis of Alzheimer's disease or mild cognitive impairment, a MMSE score between 12 and 26, a Modified Hachinski Ischemia Scale score of less than 5, a short form Geriatric Depression Scale score of less than 7, a positron emission tomography (PET) scan confirming amyloid plaque load using 18F-Florbetapir, and the ability to provide informed consent. FUS treatments in AD patients was carried out using a single-element FUS transducer (H-231, Sonic Concepts, Bothell, WA, USA) with a passive cavitation detector (PCD) confocally aligned with the FUS transducer. A target and trajectory in the prefrontal cortex were selected by a neurologist prior to treatment, and used in numerical simulations (K-Wave Matlab Toolbox) to estimate the skull insertion loss 80,81. A neuronavigation system (Brainsight, Rogue Research, Montreal, QC, Canada) was used during treatment to align the transducer along the preplanned trajectory. FUS treatment lasted for 2 minutes, where the FUS transducer was driven at a center frequency of 0.25 MHz, with a pulse length of 10 ms, pulse repetition frequency of 2 Hz, and an estimated derated peak-negative pressure of 200 kPa, corresponding to a mechanical index of 0.4. A bolus injection of microbubbles (Definity®, Lantheus, Billerica, MA, USA) at the clinically approved dose of 0.1 mL/kg was delivered at the start of the sonication. Immediately following FUS treatment, MRI was performed with and without MR-contrast agent to assess safety and efficacy. Safety scans were first performed without contrast and include a T2-weighted-fluid-attenuated inversion recovery (FLAIR) and diffusion-weighted imaging (DWI) sequences. Afterwards, a contrast-enhanced T1-weighted MRI scan (0.2 mL/kg intravenous Dotarem) was performed to detect the location and volume of the BBB opening. T1-weighted scans were acquired approximately 15 - 20 minutes after contrast injection. For the assessment of amyloid beta load, a baseline amyloid PET was performed 2 months before treatment, with 2 follow-up PET scans acquired 3 weeks and 3 months after treatment. The subject was injected with 10mCi of 18F-Florbetapir (Amyvid®, PETNET) approximately 30 minutes before the scan in a Biograph 64 PET/CT scanner (Siemens, Munich, Germany). CT correction was applied to the raw scan to account for differences in the photon attenuation coefficient (CT scan resolution: 0.59 mm × 0.59 mm × 4 mm). The in-plane resolution of the PET scan was 1 mm, and the slice thickness was 4 mm. To analyze the amyloid changes after treatment, the PET scans were first registered to the Montreal Neurological Institute (MNI) space 82 and segmented with the Automated Anatomical Labeling 2 83,84 using software packages Clinica 85, SPM 86, and PETPVC 87. The standard uptake value ratio (SUVr) map from each scan was then quantified by normalizing with the average PET signal of the whole cerebellum as reference region. Finally, percent changes of SUVr in the frontal lobes of the 2 post-FUS scans compared to the baseline scan were calculated to assess the amyloid load change following treatment.

### Statistical analysis

All values are expressed as means ± standard error of the mean (SEM). Longitudinal analysis of the BBB opening volume was analyzed with multiple Student's t tests. Differences between the means of the sham and treated groups were analyzed using Student's t tests while 2-way repeated measures ANOVA was employed for the MWM analysis. Statistical analysis was performed on the mean value obtained per animal and the P values in the analyses were adjusted based on the Holm Sidak post hoc correction when necessary. All statistical analyses were performed using Prism 8 (Graphpad Software, San Diego, CA, USA) and the null hypothesis was rejected at the 0.05 level. Throughout the manuscript the “F” value in respect to the associated degrees of freedom is provided with the P value adjusted to the corrected multiple t-tests. The significance levels correspond to: * P < = 0.05, ** P < = 0.01, *** P < = 0.0005, **** P < 0.0001.

## Supplementary Material

Supplementary figure.
