# Supplementary material for: Focused ultrasound mitigates pathology and improves spatial memory in Alzheimer's mice and patients
Source: Theranostics. 2023 Jul 14;13(12):4102–20. doi: 10.7150/thno.79898 (PMC10405840; doi:10.7150/thno.79898)
Supplement: Supplementary file 1 — Supplementary figure. [file thnov13p4102s1.pdf]

Supplementary Material

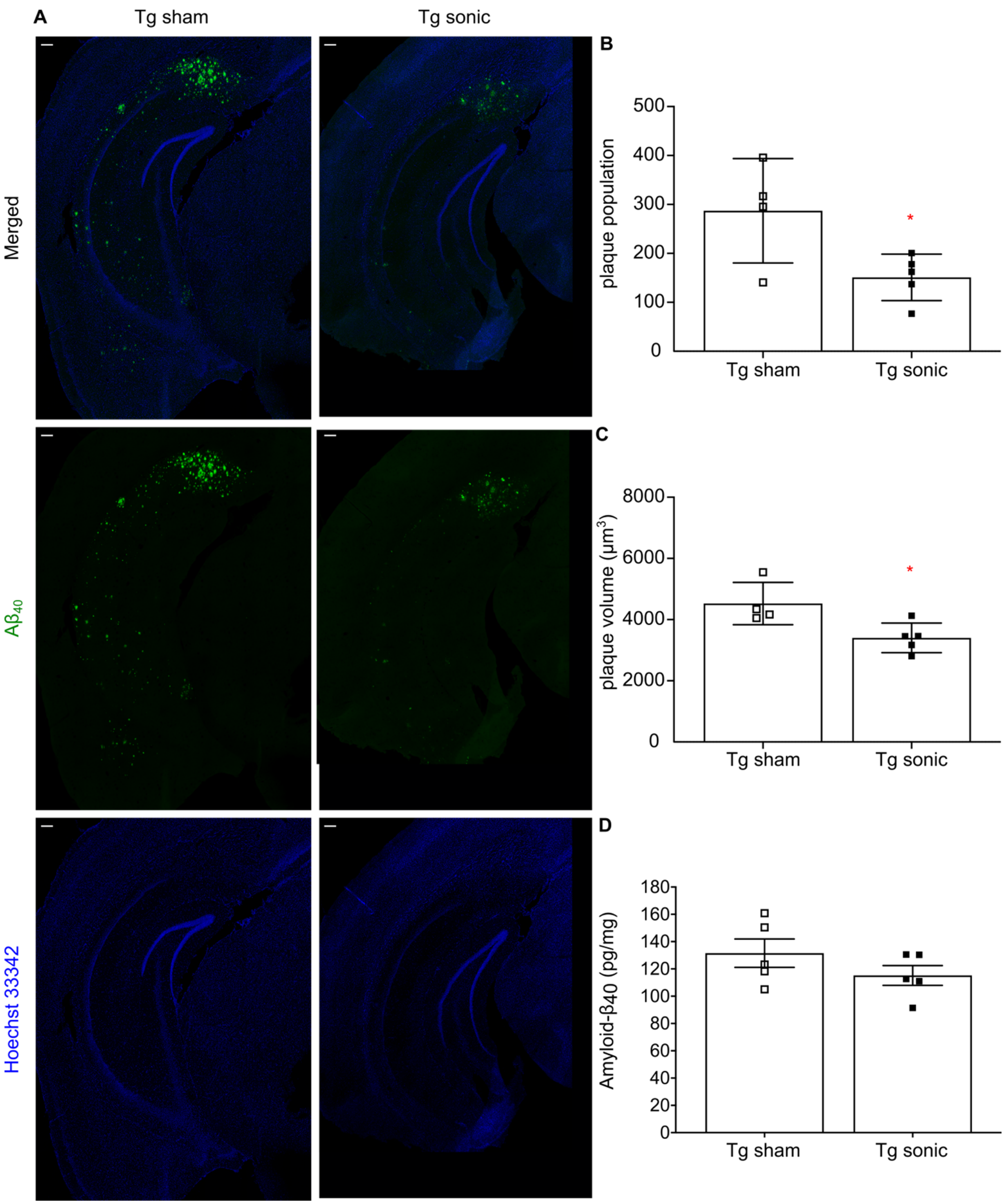

Supplementary Figure 1: Amyloid plaque quantification. A. Aβ immunoreactivity was examined using the Aβ40 specific antibody used in both immunohistochemistry and ELISA while Hoechst 33342 was employed for anatomical navigation. B. Plaque quantification revealed a decrease in the population and volume on the order of 47.41% ($t[7]=2.584$; $P=0.0363$) and 24.79% ($t[7]=2.871$; $P=0.024$), respectively, in the sonicated brains. Along the same lines, Aβ40 quantification with sandwich ELISA confirmed the decreasing trend following sonications by 12.42%, yet lacking significance ($t[9]=1.289$; $P=0.2335$). Scale bar: 100μm.
